# Research on Diamond Nano-Grinding of 4H-SiC Crystals and Wear of Abrasives with Different Sharpness

**DOI:** 10.3390/mi17040442

**Published:** 2026-04-01

**Authors:** Lijie Wu, Song Fan, Hanxiao Li, Zijuan Han, Ping Yang, Xiuting Zhao, Jisheng Pan

**Affiliations:** 1 School of Electromechanical Engineering, Guangdong University of Technology, Guangzhou 510006, China; wulj115086@hanslaser.com (L.W.);; 2 State Key Laboratory for High Performance Tools, Guangdong University of Technology, Guangzhou 510006, China; 3 School of Advanced Manufacturing, Guangdong University of Technology, Jieyang 522000, China

**Keywords:** 4H-SiC, grinding speed, subsurface damage abrasive wear, graphitization

## Abstract

Single-crystal 4H-SiC, as a wide-bandgap semiconductor material, has become a key substrate for high-power electronics and radio frequency devices due to its outstanding characteristics such as high-voltage tolerance, high-temperature stability, high-frequency efficiency and low loss. However, its inherent properties of high hardness and low fracture toughness also pose severe challenges to the ultra-precision processing of wafer substrates. In this study, through molecular dynamics methods, the influence of diamond abrasive grains with different sharpness on the processing of 4H-SiC at different grinding speeds was simulated, with a focus on analyzing its surface morphology, material removal behavior and subsurface damage characteristics. The structural evolution of 4H-SiC workpieces and diamond abrasive grains was identified through the radial distribution function, and the dynamic changes in temperature and stress during processing were further investigated to clarify the mechanism of abrasive wear and graphitization phenomena. The results show that regular octahedral abrasive grains with higher sharpness have better material removal efficiency, but they also cause more significant subsurface damage. Increasing the grinding speed helps to reduce the depth of subsurface damage. In addition, high temperature and high stress are the key factors leading to the transformation of diamond into graphite. Even under low-speed grinding conditions, the edges of the abrasive grains may still undergo graphitization due to stress concentration. The above findings have theoretical significance for an in-depth understanding of the material removal mechanism of 4H-SiC nano-grinding, and can also provide an important reference for the development of high-performance grinding wheels for SiC grinding.

## 1. Introduction

Single-crystal SiC, as a typical representative of third-generation semiconductor materials, is increasingly attracting much attention from both the academic and industrial communities. It features a wide bandgap, high breakdown field strength and excellent chemical stability [1,2,3], which enables it to exhibit outstanding physical and electrical properties under extreme working conditions such as high temperature, high pressure, high frequency and high power, significantly outperforming traditional semiconductor materials. Therefore, single-crystal 4H-SiC has become a key substrate material for the development of high-power-density and high-integration electronic devices [4]. However, the inherent mechanical properties of this material, such as high hardness, high brittleness and low fracture toughness, also pose severe challenges to its precision processing. During the processing, surface and subsurface brittle damage is prone to occur, which not only seriously affects the mechanical integrity and electrical performance of the wafer, but also significantly shortens the service life of the device [5]. In addition, to meet the performance requirements of high-frequency and high-power devices, the surface of SiC wafers often needs to achieve an ultra-high finish close to a mirror surface and sub-nanometer surface shape accuracy [6]. This further increases the difficulty of ultra-precision machining. Therefore, how to achieve low-damage and high-precision processing of single-crystal 4H-SiC, while ensuring high material removal efficiency, has become a key technical challenge that must be overcome to promote its large-scale application in next-generation semiconductor devices.

During the grinding process of 4H-SiC, cracks will be induced to form and propagate on its surface due to combined mechanical actions such as the extrusion, scraping and plowing of diamond abrasive grains, and then structural damage will occur in the surface layer and subsurface layer of the workpiece. Because this process occurs at the micro–nanoscale and is extremely transient, traditional experimental methods find it difficult to directly observe and verify its mechanism. Against this backdrop, molecular dynamics simulation has emerged as an effective means for in-depth exploration of the material removal mechanism and microstructure evolution during ultra-precision grinding [7]. Using the MD method, several studies have focused on the influence of different factors on the grinding behavior of 4H-SiC. For instance, Chen et al. [8] compared the differences in grinding force and subsurface damage between 3C-SiC and 4H-SiC under different grinding parameters; Wang et al. [9] combined MD simulation with nanoindentation experiments to analyze the influence of defects on the damage behavior of materials and found that the two were consistent in force–displacement response. Ni et al. [10] studied the nanoindentation and scratch responses of 4H-SiC in different crystal orientations from the perspective of crystal anisotropy, and pointed out that the lattice damage caused by scratches along the [$1\bar{1}00$] direction was relatively small. At present, the molecular dynamics research on 4H-SiC mainly focuses on the plastic removal behavior of the material, the crystal orientation effect, and the influence of tool geometric parameters, etc. [11,12,13]. In these simulations, given that the hardness and elastic modulus of diamond are much higher than those of 4H-SiC, diamond abrasive grains are usually simplified as ideal rigid bodies that do not deform. Although this assumption improves the computational efficiency, it also ignores the wear and structural evolution of the abrasive grains themselves during processing to a certain extent.

Grinding speed, as a key process parameter, has a significant impact on the material removal mechanism [14]. Regulated by the strain rate effect, grinding speed not only significantly affects the deformation and damage behavior of the workpiece material, but also profoundly influences the wear process of the abrasive and the graphitization transformation of diamond. Existing studies have explored the mechanism of grinding speed in precision machining from different perspectives: Zhang et al. [15] investigated the influence of strain rate on the deformation and friction behavior of 4H-SiC through high-speed scratch experiments and found that a higher scratch speed was more likely to introduce surface defects and promote the ductile–brittle transition at a shallower scratch depth. Wang et al. [16] further investigated the influence of scraping speed on the phase transformation behavior of single-crystal silicon during high-speed scratching. The results showed that as the scraping speed increased, the residual depth of the scratch decreased, while the degree of phase transformation correspondingly enhanced. The above achievements consistently indicate that grinding speed plays an indispensable regulatory role in the removal mechanism and damage evolution of brittle solid materials. However, current research mostly focuses on the influence of process parameters on the behavior of workpieces. When processing 4H-SiC with diamond abrasives of different sharpness at different grinding speeds, there is still a lack of systematic and in-depth discussions on how the wear evolution and graphitization phenomenon of the abrasives themselves further affect the removal behavior and surface quality of the materials.

This study takes the nanoscale grinding process of 4H-SiC crystals as the object and systematically explores the material removal mechanism, the wear behavior of diamond abrasive grains and the graphitization transformation phenomenon. For this purpose, a molecular dynamics model of the nano-grinding of diamond abrasive grains with different sharpness was established, and the processing procedures at various grinding speeds were simulated. The research focuses include: the influence of grinding speed on subsurface damage of workpieces, material removal methods and structural phase transformation; the distribution and evolution of temperature and stress during grinding; the wear characteristics and graphitization behavior of abrasive grains with different sharpness levels; and the variation law of the surface roughness of the workpiece. The research results are expected to deepen the understanding of the damage evolution and removal mechanism of 4H-SiC at the nanoscale, and at the same time, reveal the influence law of grinding speed on the wear and graphitization of diamond abrasive grains. In addition, this research can also provide theoretical references and process guidance for the high-precision and low-damage processing of brittle solid materials.

## 2. Simulation Methods and Conditions

During the grinding process of 4H-SiC crystals, the interaction between the workpiece and the diamond abrasive is extremely complex, and it is difficult to directly establish the quantitative relationship between grinding process parameters and subsurface damage through traditional experimental means. In contrast, molecular dynamics simulation methods can capture the transient evolution details of damage formation and reproduce the entire process of crack initiation and propagation at the atomic scale. This study is based on molecular dynamics simulations of a single diamond abrasive. It systematically constructs and analyzes the process of diamond abrasive nanomilling of single-crystal 4H-SiC. This model can eliminate the random interference among multiple abrasive grains, thereby clearly revealing the intrinsic relationship between the geometric features of a single abrasive particle, its motion parameters, and the material response. However, this simplification also leads to the difficulty in fully replicating the superimposed stress field formed by multiple abrasive grains, the interaction and competition of chips between abrasive grains, and the homogenizing effect of the random distribution of abrasive grains on the surface morphology in actual milling. Using this model, this paper focuses on studying the influence laws of different sharpnesses of diamond abrasive particles on the material removal behavior, surface morphology, subsurface damage, crystal structure transformation, abrasive wear behavior, and interface tribological properties of 4H-SiC workpieces at different milling speeds.

**Figure 1:**
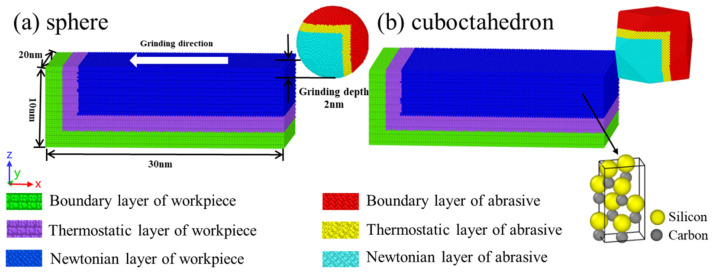
Model of diamond nano-ground single-crystal 4H-SiC. (**a**) sphere; (**b**) cuboctahedron.

Molecular dynamics simulation reveals the evolution law of the system over time from a dynamic perspective by tracking the movement trajectories of atoms and molecules within a given time range [17]. In this study, LAMMPS was used to construct a simulation model of diamond nano-grinding single-crystal 4H-SiC. The model structure is shown in Figure 1, and OVITO 3.10.6 was used to conduct post-processing and visual analysis of the simulation process and results. This model is mainly composed of two parts: diamond abrasive grains and single-crystal 4H-SiC workpieces. Figure 1a and Figure 1b respectively show the geometric configurations of spherical abrasive grains and regular octahedral abrasive grains. To strike a reasonable balance between simulation accuracy and computational efficiency, the selection of workpiece size should follow the following principles: on the one hand, it should be large enough to mitigate the size effect that may occur at the nanoscale; on the other hand, the number of atoms should also be moderately controlled to reduce the computational burden [18]. Based on this, in this model, the 4H-SiC workpiece is designed as a cuboid structure, with dimensions of 30 nm, 20 nm, and 10 nm along the X, Y, and Z directions, respectively, and contains a total of 591,920 atoms. To effectively simulate the thermodynamic coupling effect in the actual grinding process and maintain system stability, the workpiece is divided into a three-layer structure during modeling, from bottom to top: the boundary layer, the constant-temperature layer, and the Newton layer. Among them, the boundary layer atoms remain stationary, which is used to constrain the overall displacement of the workpiece, suppress the boundary effect and maintain the structural symmetry of the crystal. The constant temperature layer adopts the isothermal and isobaric system (NVT) and temp/rescale temperature control method to keep the temperature in this area at 300 K all the time, so as to reasonably dissipate the heat generated during the grinding process and avoid the accumulation of system temperature. The Newton layer employs a micro-canonical ensemble (NVE), and the atoms follow the classical Newton’s laws of motion. Due to the introduction of internal stress by setting different atomic layers, before conducting grinding simulation, the workpiece model needs to be fully relaxed through the conjugate gradient algorithm until the system energy converges to the minimum value, in order to obtain a stable initial structure. Periodic boundary conditions are set in the Y direction to simulate the “infinite width” feature of the workpiece in this direction, effectively suppressing boundary effects and enhancing the consistency between the model and the actual continuous material behavior [19,20]. In the X and Z directions, contraction boundary conditions are adopted to reasonably limit the degrees of freedom of the chips and prevent non-physical self-overlapping when crossing the boundary, and thereby more truly reflect the chip formation and material deformation behavior.

**Figure 2:**
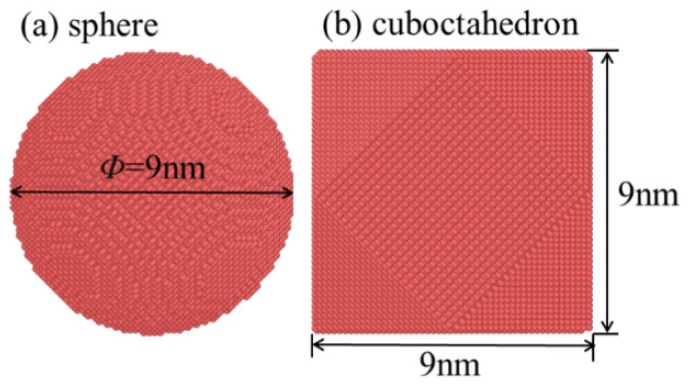
Shape and size of diamond abrasive grains. (**a**) sphere; (**b**) cuboctahedron.

To systematically explore the wear behavior of diamond abrasives with different sharpness at various grinding speeds, this study set the diamond abrasives as a non-rigid body model capable of deformation. Its specific geometric shape and dimensions are shown in Figure 2. At the initial moment of the simulation, a gap of 1 nm was maintained between the diamond abrasive and the workpiece surface in the X direction. The abrasive was located at the center of the workpiece in the Y direction, while its position in the Z direction was determined by the grinding depth. In this study, the grinding depth was uniformly set at 2 nm. Based on the commonly used process parameter ranges in relevant grinding experiments and simulation literature [21,22], this study selected four grinding speeds of 50 m/s, 100 m/s, 200 m/s, and 400 m/s for simulation analysis. Although the adopted grinding speeds are higher than the typical speeds in actual 4H-SiC grinding processes, this difference mainly stems from the fact that the simulation needs to apply a sufficiently high strain rate within an extremely short nanosecond time window at the atomic scale, in order to effectively stimulate the plastic deformation, dislocation evolution, and phase transformation behaviors of the material with limited computing resources, thereby revealing its microscopic removal mechanism and damage laws. The specific simulation parameter settings are summarized in Table 1.

Furthermore, the reliability of the molecular dynamics simulation results largely depends on the accuracy of the interatomic potential functions adopted. In this study, the interaction between diamond abrasives and 4H-SiC workpieces involves three types of atomic pairs: C-C, Si-Si and C-Si. Studies have shown that the Tersoff potential function can describe the interaction forces of these covalent bond systems [23,24] well, and it is particularly suitable for accurately characterizing the material removal behavior and interface response of diamond abrasives and 4H-SiC crystals during the nano-grinding process [25].

## 3. Results and Discussion

The sharpness of the abrasive and the grinding speed are the key process parameters that affect the material removal efficiency and the surface/subsurface integrity of the workpiece [26]. To deeply reveal its mechanism of action on the processing procedure, this section systematically analyzes the material deformation and removal behavior, subsurface damage distribution, evolution of the stress field and temperature field, abrasive wear characteristics and grinding force variation law of 4H-SiC workpieces under different sharpness abrasives and multiple sets of grinding speeds. The research results aim to provide theoretical support for optimizing the process parameters of 4H-SiC ultra-precision grinding and achieving high-efficiency and low-damage processing.

### 3.1. Surface Morphology

**Figure 3:**
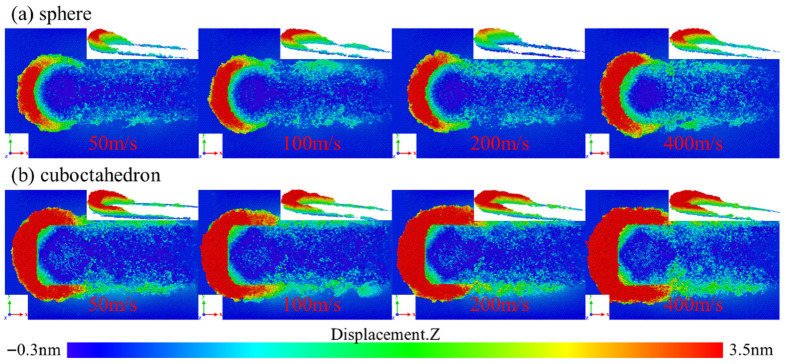
Surface morphology and chip distribution of the workpiece at different grinding speeds. (**a**) sphere; (**b**) cuboctahedron.

During the nano-grinding process, the 4H-SiC workpiece is subjected to the shearing and extrusion of diamond abrasive grains. The atomic movement mainly manifests in two forms: some atoms are stripped along the direction of abrasive grain movement to form chips, while the other part migrates to both sides of the grinding groove and accumulates. To systematically compare the influence of diamond abrasive grains with different sharpness on the material removal behavior of 4H-SiC workpieces at different grinding speeds, in this study, the simulation results were post-processed based on the Ovito 3.10.6 software, and the coloring characterization was carried out according to the displacement values of each atom in the Z-axis direction relative to the initial position. The surface morphology and chip distribution of 4H-SiC workpieces under different grinding speeds are shown in Figure 3. The color of atoms in the figure reflects the change in their distance from the original surface, thereby visually revealing the microscopic characteristics of material removal and surface evolution.

It can be clearly observed from Figure 3 that under the grinding depth condition of 2 nm, both the spherical abrasive grains with a smooth surface and the regular octahedral abrasive grains with sharp vertices and edges form obvious grinding grooves on the surface of the 4H-SiC workpiece. These grooves directly reflect the cutting effect of the abrasive grains on the workpiece, indicating that both types of abrasive grains have obvious material removal capabilities.

**Figure 4:**
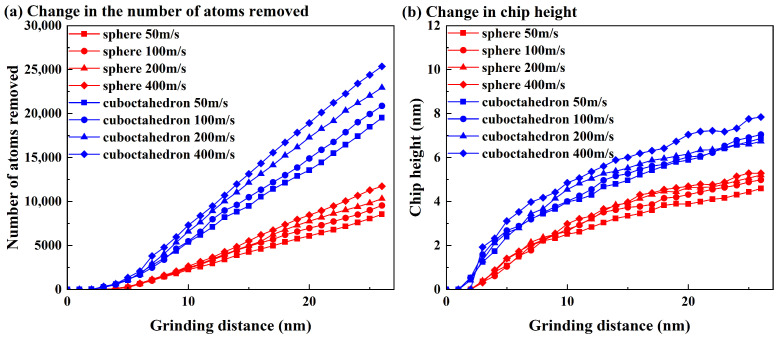
Number of removed atoms and height of chip accumulation at different grinding speeds. (**a**) Change in the number of atoms removed; (**b**) Change in chip height.

To further quantify the morphological features of the workpiece surface after grinding, this study characterized the material removal behavior by counting the number of chip atoms and the height of chip accumulation. The results are shown in Figure 4. The red and blue curves in the figure correspond respectively to the processing effects of spherical abrasives and regular octahedral abrasives, and different symbol marks indicate different grinding speeds. As can be seen from Figure 4, with the increase in grinding distance and grinding speed, the number of chips and the stacking height produced by the two abrasives both show an upward trend, and the octahedral abrasive is significantly higher than the spherical abrasive in both indicators. Meanwhile, the grinding speed has a more significant influence on octahedral abrasives. The increase in the number of chips and the stacking height with the increase in speed is significantly greater than that of spherical abrasives. This difference mainly stems from the geometric characteristics of regular octahedral abrasives. Its sharp vertices and edges are prone to cause local stress concentration and impact effects during grinding, thereby promoting material peeling. In addition, its geometric shape also hinders the smooth discharge of chips to both sides, further promoting the accumulation of chips in front of the grinding groove. Especially under high-speed grinding conditions (200 m/s and 400 m/s), stress concentration and impact effects are more prominent, making it easier for regular octahedral abrasives to form deep grooves and steep side walls. It is worth noting that as can be seen from Figure 4b, in the initial stage of grinding, the chip accumulation height rises rapidly, but as grinding proceeds, the growth rate gradually slows down. This phenomenon can be attributed to two aspects. First, the abrasive grains wear out during continuous grinding, and their cutting capacity gradually decreases. Second, the increase in the accumulation of chips in front of the abrasive grains leads to an increase in movement resistance, thereby inhibiting the further growth of the accumulation height.

**Figure 5:**
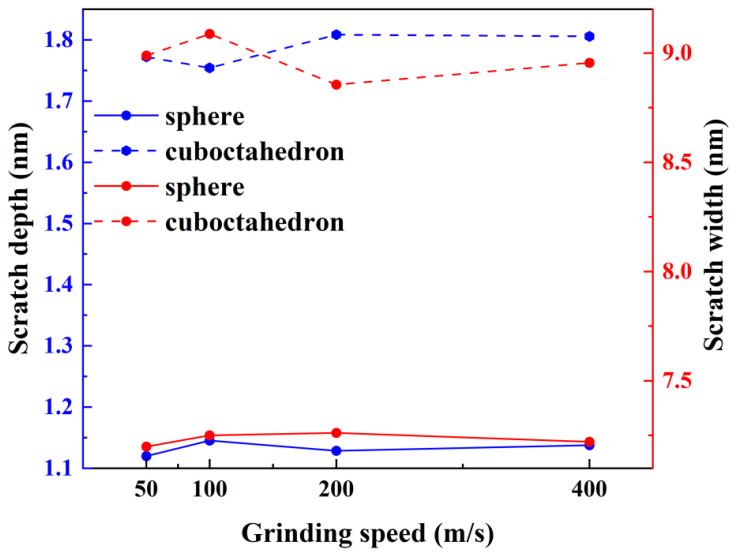
Influence of grinding speed on scratch depth and scratch width.

To deeply analyze the influence of abrasive grains with different sharpness on the surface morphology of workpieces at different grinding speeds, this study quantitatively measured the depth and width of surface scratches after grinding. The results are shown in Figure 5. The solid and dashed lines in the figure represent the processing results of the spherical abrasive and regular octahedral abrasive, respectively, while the blue and red lines correspond to the measured values of scratch depth and width, respectively. It can be seen from Figure 5 that the influence of grinding speed on the depth and width of scratches is relatively limited. In contrast, the influence of abrasive sharpness is more significant: the scratches produced by regular octahedral abrasives are significantly greater in both depth and width than those of spherical abrasives. Further observation of the grinding process reveals that only at the initial contact stage of the work, the actual scratch size is close to the preset grinding depth and abrasive grain width. After entering the stable processing stage, due to abrasive wear and the elastic recovery effect of the workpiece material, the actual scratch size is generally smaller than the preset value, especially in the processing of spherical abrasives. Data shows that at the end of grinding, the scratch depth formed by the spherical abrasive is only about 57% of the preset grinding depth, and the scratch width is about 80% of the preset abrasive grain width.

Based on the above analysis, it can be known that the sharpness of abrasive grains and grinding speed significantly affect the surface morphology of 4H-SiC workpieces. The sharp octahedral abrasive grains with high sharpness exhibit higher material removal efficiency due to their sharp geometric features, and this efficiency is further amplified with the increase in grinding speed. However, high-efficiency removal may also be accompanied by deeper grooves and steeper side wall structures, which pose higher requirements for surface integrity. The results of this study have significant guiding significance for optimizing the grinding process of 4H-SiC: By rationally selecting the shape of abrasive grains and regulating the grinding speed, a better balance can be achieved between the material removal rate and the surface quality, providing a theoretical basis for realizing high-efficiency and low-damage ultra-precision processing.

### 3.2. Analysis of Subsurface Damage

**Figure 6:**
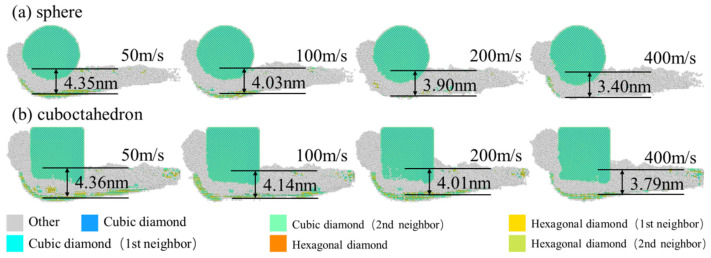
Morphology of subsurface damage at different grinding speeds. (**a**) sphere; (**b**) cuboctahedron.

Subsurface damage depth is a key indicator for evaluating the integrity of the machined surface of a workpiece. It reflects the changes in the microstructure and defect levels beneath the surface layer of the workpiece during the machining process, and usually involves a series of physical phenomena such as amorphous and crack formation. During the grinding process of 4H-SiC with diamond abrasives, sub-surface damage is mainly induced by mechanical actions (such as extrusion, scraping and plowing) and frictional thermal effects. These coupled actions promote local plastic deformation and structural damage of the material [27,28]. 4H-SiC, as a typical hard and brittle material, is prone to subsurface damage during processing, thereby affecting the service life and performance of devices. The subsurface damage zone is located beneath the wear mark, and its damage depth is defined as the normal distance from the upper surface to the deepest amorphous atoms of the workpiece after elastic recovery. The sub-surface damage caused by abrasive particles of different sharpness at different grinding speeds to the 4H-SiC workpiece is shown in Figure 6. The results show that both the shape of the abrasive grains and the grinding speed have an impact on the depth of subsurface damage. At the bottom of the damaged area, significant structural phase transitions can be observed, with the appearance of crystal phases such as hexagonal diamond and cubic diamond, and the atomic lattice in the processing area has undergone severe distortion, gradually transforming into amorphous structures existing on the processing surface and in the chips. Meanwhile, diamond abrasives also show obvious amorphous phenomena during the wear process.

**Figure 7:**
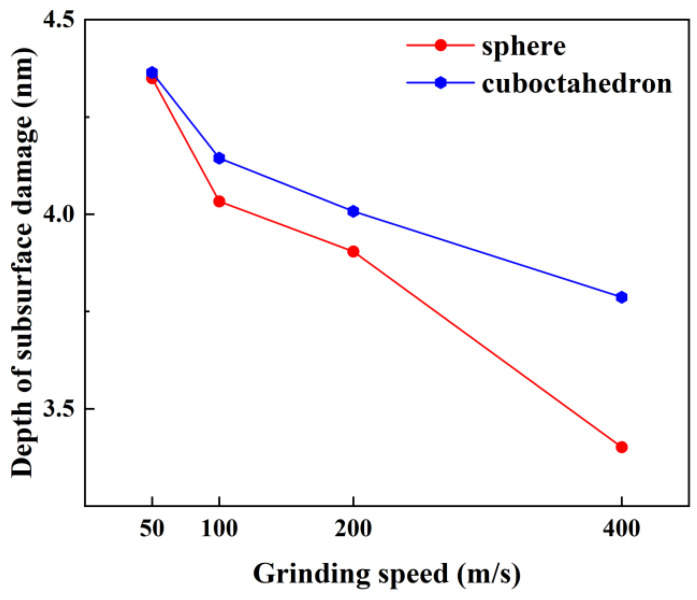
Influence of grinding speed on the depth of subsurface damage.

The depth of subsurface damage caused by abrasive materials of different sharpness under different grinding speeds on the 4H-SiC workpiece is shown in Figure 7. It can be clearly observed that the octahedral abrasive with higher sharpness causes a greater depth of subsurface damage compared to the spherical abrasive. In addition, as the grinding speed increases, the depth of subsurface damage caused by both abrasives gradually decreases. Specifically, the depth of subsurface damage caused by spherical abrasives was significantly reduced from 4.349 nm at a grinding speed of 50 m/s to 3.401 nm at 400 m/s, with a reduction rate as high as 21.8%. In contrast, the subsurface damage depth caused by the regular octahedral abrasive with higher sharpness decreased from 4.364 nm at a grinding speed of 50 m/s to 3.787 nm at 400 m/s, with a reduction rate of 13.2%. This phenomenon can be explained by the “skin effect” based on dislocation dynamics and crack propagation mechanisms [29].

Increasing the grinding speed can effectively promote the material removal of 4H-SiC in the ductile mode, which is helpful to suppress subsurface damage and improve the surface quality of the processed material. As the grinding speed increases, the strain rate also increases accordingly. Under high strain rate conditions, the atoms within the material do not have sufficient time to complete crack propagation. The stress release is more likely to be achieved through short-range plastic mechanisms such as dislocation slip and the formation of dislocation loops. At this time, the driving force for dislocation movement increases, and the dislocation slip rate and phase transformation rate also rise, thereby alleviating the local stress concentration and reducing the probability of brittle damage. In addition, the increase in grinding speed intensifies the relative motion between the abrasive and the workpiece, not only accelerating the heat generation rate and increasing the frictional heat, but also significantly shortening the heat diffusion time, resulting in a rapid rise in the local temperature of the grinding area. The heat is mainly concentrated on the surface layer of the workpiece and the chip area, causing a significant increase in grinding temperature. An increase in temperature leads to a decrease in the hardness and yield strength of materials and an increase in their plasticity, thereby promoting local ductile deformation and suppressing brittle fracture. From an atomic-scale perspective, the increase in grinding speed enhances the thermal vibration of atoms, weakens the bonding strength between atoms, and lowers the lattice barrier. Under this condition, stressed atoms are more likely to release energy through plastic slip rather than cleavage fracture. The above-mentioned mechanisms jointly promote the removal of the material in a dislocation-dominated ductile manner, while hindering the extension of brittle cracks to the subsurface layer, thereby effectively reducing the depth of subsurface damage. The research results show that appropriately increasing the grinding speed has a positive effect on inhibiting the propagation of subsurface damage.

**Figure 8:**
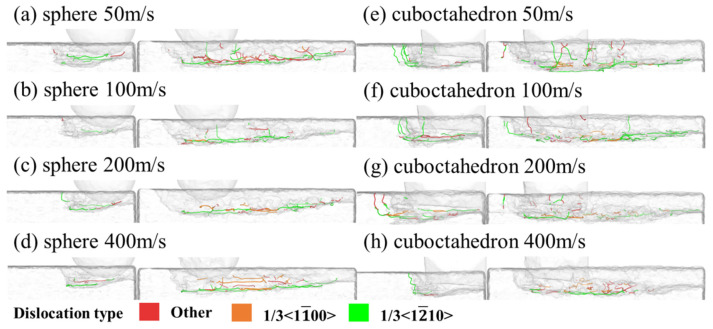
Evolution process of subsurface dislocations of abrasives with different sharpness at different grinding speeds.

**Figure 9:**
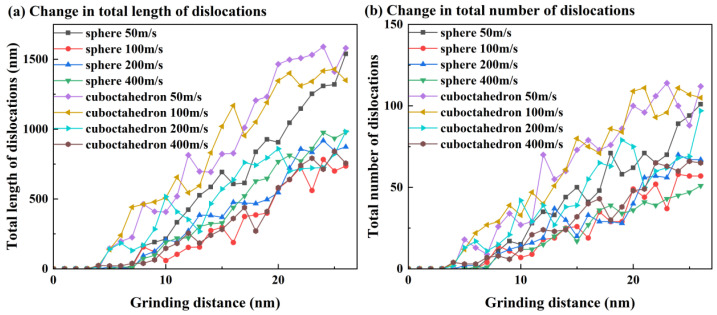
Variations in the total length and quantity of dislocations of abrasives with different sharpness levels at different grinding speeds. (**a**) Change in total length of dislocations; (**b**) Change in total number of dislocations.

Different abrasive materials with different sharpness are shown in Figure 8 under different grinding speeds: the sub-surface dislocation evolution process of the 4H-SiC workpiece material at two typical moments when the abrasive completely enters the grinding zone, and at the end of the simulation. The main observed dislocation types include 1/3<$1\bar{1}00$>, 1/3<$1\bar{2}10$> and other categories, and these dislocations are mainly concentrated in the bottom area of the subsurface damage layer. Figure 9 further presents the variation patterns of the total length and quantity of dislocations of abrasives with different sharpness levels at different grinding speeds. It can be clearly seen that with the increase in grinding speed, the total length and quantity of dislocations show a downward trend. This phenomenon may stem from the following mechanism: Under high-speed grinding conditions, the loading rate is relatively high, and the atomic response time is insufficient, which hinders the full nucleation and slip of dislocations. Meanwhile, the local temperature rise caused by high-speed grinding promotes the phase transformation and amorphous transformation of the material, further inhibiting the generation and expansion of dislocations, thereby leading to a decrease in dislocation density and total length.

In addition, it can be observed that at a lower grinding speed, the total length and quantity of dislocations show a relatively stable upward trend as the grinding distance increases. However, at higher grinding speeds, the fluctuations during the growth process of both are significantly intensified, and the fluctuation amplitude and frequency caused by the regular octahedral abrasive grains with higher sharpness are more pronounced. This phenomenon can be explained from the following perspective: Under low-speed grinding conditions, the stress distribution on the material is relatively uniform, and the process of dislocation nucleation and accumulation is relatively continuous and stable, which promotes a steady increase in dislocation density and total amount. When the grinding speed increases, the frictional heat in the grinding zone significantly rises. The local temperature increase leads to a decrease in material hardness and an increase in plasticity, thereby affecting the generation and movement behavior of dislocations. Meanwhile, the sharp geometric features of the regular octahedral abrasive particles can easily cause intense stress concentration on the workpiece surface. Although this effect may promote the nucleation and proliferation of dislocations, it also leads to the rapid accumulation or annihilation of dislocations locally, causing significant fluctuations in the total amount and density of dislocations.

In conclusion, grinding speed is a key process parameter for regulating the subsurface damage of 4H-SiC workpieces, directly affecting the material removal mechanism and the final processing quality. By rationally optimizing the grinding speed, the depth of subsurface damage can be effectively controlled, thereby significantly improving the surface integrity of the workpiece and prolonging its service life. A thorough understanding of the effect law of grinding speed on material removal behavior is of great engineering guiding significance for optimizing grinding processes, improving processing efficiency and ensuring surface quality.

### 3.3. Temperature and Stress

Grinding temperature and stress distribution are two key physical fields that characterize energy conversion and interface mechanical behavior during the grinding process [30]. Grinding temperature reflects the accumulation and dissipation of thermal energy in the processing area, directly affecting the local softening and phase transformation behavior of the material. The stress distribution reveals the micro-force characteristics between the abrasive and the workpiece, which is crucial for clarifying the mechanism of plastic deformation and brittle fracture at the nanoscale. Therefore, this section will systematically explore the influence laws of different sharpness abrasives and different grinding speeds on the distribution of the grinding temperature field and stress field.

Temperature, as a physical quantity for measuring the intensity of atomic thermal motion, reflects the mutual conversion process of atomic kinetic energy, chemical energy and thermal energy within the system [31,32]. During the grinding process of 4H-SiC, the breaking and recombination of covalent bonds can cause local temperature changes, which in turn affect the material removal behavior, surface/subsurface damage characteristics, and abrasive wear state. It is one of the key factors determining the processing quality of workpieces and the service life of abrasive grains [33,34].

**Figure 10:**
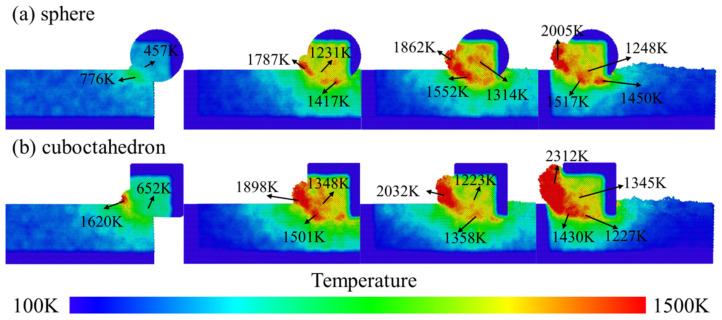
Temperature changes in abrasives with different sharpness levels at a grinding speed of 100 m/s. (**a**) sphere; (**b**) cuboctahedron.

When the grinding speed was 100 m/s, the evolution of the temperature field distribution caused by different sharpness levels of diamond abrasives on the 4H-SiC workpiece is shown in Figure 10. The figure compares the influence of two abrasive shapes, spherical and regular octahedral, on the surface and near-surface temperature of the workpiece. During the grinding process, the mechanical action of abrasive grains and the interfacial friction effect cause a large amount of heat to accumulate in the grinding area, resulting in local high temperatures. This local temperature rise will significantly alter the microstructure and mechanical response of the material, such as promoting dislocation movement, and inducing structural phase transformation and amorphous phenomena, thereby directly affecting the surface quality and subsurface integrity of the workpiece.

To more intuitively reveal the role of temperature in material removal and the formation of subsurface damage, in this study, the intermediate regions directly affected by abrasive grains in the model were sliced and homogenized to enhance image clarity. This area has the most concentrated defects due to its direct exposure to the mechanical action of abrasive particles, which can more clearly reflect the influence of temperature changes on the microstructure of the material. By comparing the temperature distribution under different abrasive shapes, it is found that the local temperature rise caused by the regular octahedral abrasive during grinding is more significant, which is related to its sharp geometric features and stronger local stress concentration. Specifically, when the spherical abrasive is processed in Figure 10a, the temperature distribution on the workpiece surface is relatively uniform, with the maximum temperature being approximately 2005 K. In Figure 10b, when the regular octahedral abrasive is processed, the high-temperature area is more concentrated in the chip part in front of the abrasive grains, with the maximum temperature reaching 2312 K. This temperature difference indicates that the octahedral abrasive has a stronger thermal effect on the workpiece surface, which may lead to deeper subsurface damage, consistent with the damage distribution conclusion in Figure 7.

**Figure 11:**
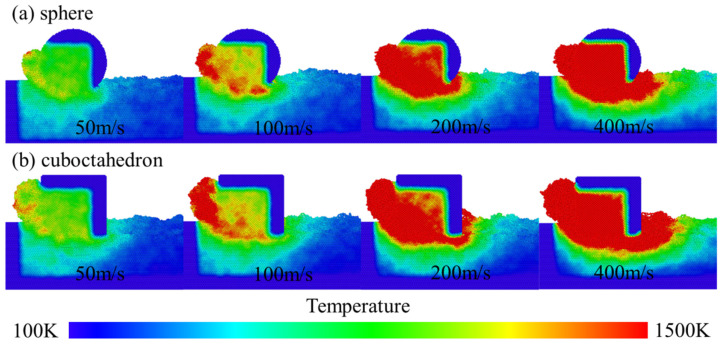
Temperature cloud map at different grinding speeds. (**a**) sphere; (**b**) cuboctahedron.

**Figure 12:**
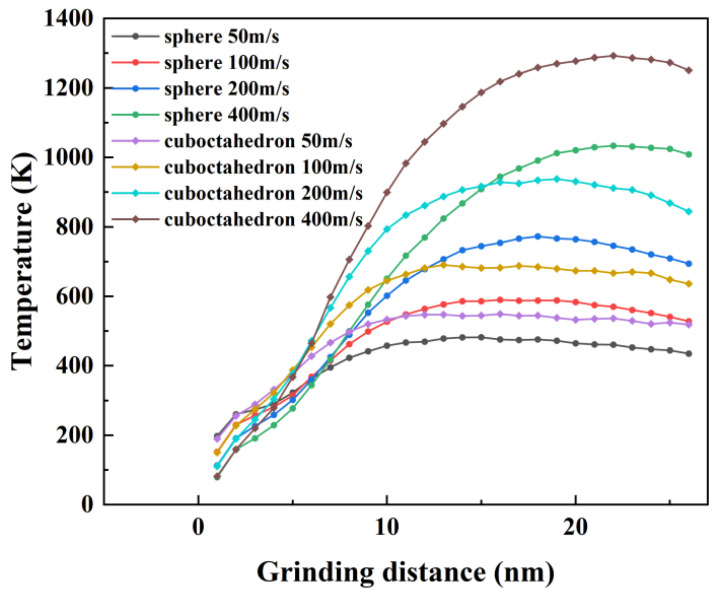
Variation in temperature with grinding distance at different grinding speeds.

The temperature field distribution during the interaction process between the two types of diamond abrasives and the 4H-SiC workpiece under different grinding speeds is shown in Figure 11. As the grinding progresses, the friction and mechanical effects between the abrasives and the workpiece generate a large amount of heat, which is mainly concentrated in the grinding layer and forms obvious temperature peaks in local areas such as the shear bands. This phenomenon is related to the short time scale of molecular dynamics simulation: the system lacks sufficient thermal diffusion time, resulting in an uneven distribution of kinetic energy and thus causing significant local temperature rise. On the other hand, the grinding contact area is relatively small, and the energy is highly concentrated at the abrasive–workpiece interface. Heat is difficult to conduct quickly to the surroundings, thus forming an instantaneous high temperature near the interface, while the temperature in the already processed area is relatively low. Grinding speed and grinding distance significantly affect energy conversion, heat generation and heat transfer mechanisms. To quantitatively evaluate the influence of process parameters, Figure 12 statistically shows the average temperature changes in the Newton layer under different grinding speeds and the action of two types of abrasives. The results show that as the grinding speed increases, the grinding temperature rises significantly, and the temperature rise amplitude caused by regular octahedral abrasives is higher than that of spherical abrasives. Meanwhile, as the grinding distance increases, the heat-affected zone gradually expands, and the overall temperature shows an upward trend. Specifically, increasing the grinding speed will intensify the friction between the rake face and the chip, as well as between the flank face and the workpiece, thereby generating more frictional heat. Under high-speed grinding, the strain rate increases, the shear deformation becomes more significant, and a larger proportion of mechanical energy is converted into thermal energy. At this point, the rate of heat generation exceeds the rate of heat dissipation, causing the local temperature to rise. In addition, during high-speed grinding, the contact time between the abrasive and the workpiece is shortened, and the diffusion of heat into the interior of the workpiece is restricted. Although the rapid separation of chips from the workpiece can carry away some heat, it further inhibits the heat transfer to the deep part of the workpiece.

von Mises stress is closely related to the plastic deformation and dislocation formation and evolution of materials. To deeply reveal the influence of stress distribution on subsurface damage, this study conducted a slice analysis of the middle region of the model and carried out homogenization treatment to enhance the visual effect of stress distribution. At a grinding speed of 100 m/s, the changes in the von Mises stress field caused by different degrees of sharpness of diamond abrasives during the grinding of 4H-SiC workpieces are shown in Figure 12. The figure compares the effects of two abrasive shapes, spherical and octahedral, on the stress distribution on the surface and sub-surface of the workpiece.

**Figure 13:**
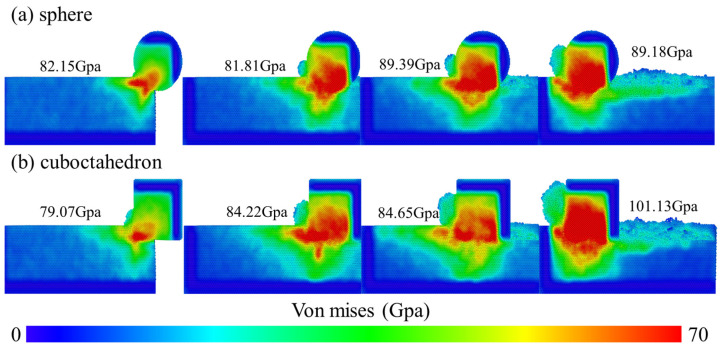
Variation in the von Mises stress of abrasives with different sharpness at a grinding speed of 100 m/s. (**a**) sphere; (**b**) cuboctahedron.

Figure 13 clearly contrasts the differences in the effects of spherical and regular octahedral abrasives on the surface stress distribution of 4H-SiC workpieces at a grinding speed of 100 m/s. At the stage when the abrasive grains initially come into contact with the workpiece surface but have not yet fully entered the grinding zone, the von Mises stress on the workpiece surface gradually increases with the increase in the contact area and contact pressure. Further observation reveals that when the spherical abrasive grains fully enter the grinding zone, the stress fluctuations they cause are relatively small, indicating that their mechanical effect on the workpiece surface is relatively stable. After the regular octahedral abrasive grains fully enter the grinding zone, the stress still continues to increase. This may be due to the significant stress concentration effect caused by the sharp vertices and edges during processing. During the entire grinding process, the peak von Mises stress generated by the spherical abrasive is relatively low, with a maximum of 89.18 GPa; the octahedral abrasive produced a higher peak stress of 101.13 GPa. This result indicates that the octahedral abrasive with high sharpness, due to its sharp geometric features, is more likely to induce plastic deformation and dislocation movement of the material during grinding, thereby forming a higher stress level on the surface layer of the workpiece.

**Figure 14:**
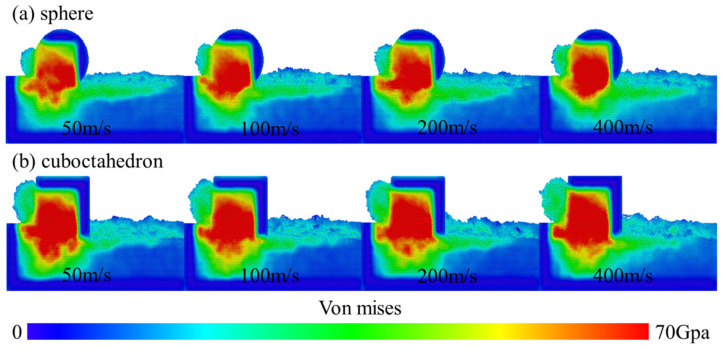
von Mises stress cloud diagram at different grinding speeds. (**a**) sphere; (**b**) cuboctahedron.

**Figure 15:**
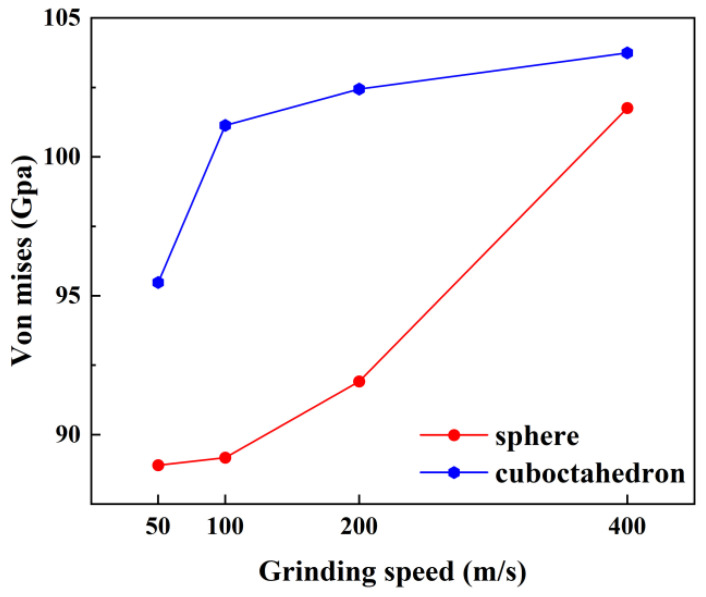
Maximum von Mises stress at different grinding speeds.

Figure 14 further presents the distribution characteristics of the stress field when the two diamond-like diamond abrasives interact with the 4H-SiC workpiece at different grinding speeds. To quantify the variation law of stress with grinding speed, Figure 15 calculates the maximum stress value after homogenization treatment. It can be seen that when the grinding speed exceeds 100 m/s, the stress change caused by spherical abrasives tends to be flat, while the stress produced by regular octahedral abrasives increases significantly. This difference mainly stems from the geometric characteristics of regular octahedral abrasive grains: their sharp vertices and edges tend to cause local stress concentration when in contact with the workpiece, enhancing the interatomic forces at the interface and thereby promoting plastic deformation of the material. In addition, the chips formed during processing are difficult to be discharged and accumulate in front of the abrasive grains, further increasing the stress in this area and transmitting it to the surrounding atoms. From an energy perspective, an increase in grinding speed means that more mechanical energy is input into the grinding zone per unit time. This part of the energy is converted into thermal energy and stress energy through friction and deformation, resulting in an increase in local temperature rise and stress. Under high-speed conditions, the increase in strain rate and the intensification of shear deformation promote more mechanical energy to be converted into thermal energy. The rate of heat accumulation exceeds the rate of heat dissipation, further pushing up the temperature and affecting the mechanical properties of the material, and intensifying stress concentration.

In conclusion, octahedral abrasives, due to their sharp geometric features, induce higher local temperatures and stresses during grinding, leading to more significant subsurface damage. As the grinding distance increases, the stress value and the range of the high-stress area gradually expand. As the grinding speed increases, the strain rate and shear deformation in the grinding zone intensify, jointly promoting a further rise in temperature and stress.

### 3.4. Structural Phase Transition

**Figure 16:**
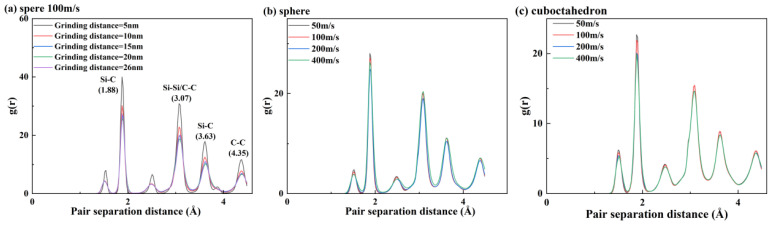
RDF curves of 4H-SiC under different grinding conditions. (**a**) sphere 100 m/s; (**b**) sphere; (**c**) cuboctahedron.

To deeply explore the structural evolution mechanism of 4H-SiC during the grinding process [35], this paper adopts a method combining the radial distribution function and crystal phase statistics for analysis. Firstly, by analyzing the peak position and intensity of the radial distribution function [36], the atomic density distribution information within a specific distance interval can be obtained. For single-crystal 4H-SiC, the following types of atomic spacings mainly exist within its unit cell: tight Si-C bonds (approximately 1.88 Å), the second-nearest Si-Si/C-C bonds (approximately 3.07 Å), the more distant Si-C bonds (approximately 3.63 Å), and the more distant Si-Si bonds (approximately 4.35 Å) are shown at the peak positions in Figure 16a.

At a grinding speed of 100 m/s, the changes in the radial distribution function corresponding to different grinding distances during the grinding of 4H-SiC material with spherical abrasive are shown in Figure 16a. It can be seen that the intensities of the characteristic peaks located at approximately 1.88 Å, 3.07 Å, 3.63 Å and 4.35 Å gradually weaken with the grinding process, indicating that the atoms within the complete lattice have shifted and the crystal structure has changed. A further comparison of Figure 16b,c shows that regardless of the sharpness of the abrasive grains, the peak strength of RDF decreases as a whole with the increase in grinding speed, indicating that a higher grinding speed will lead to more chemical bond breaks, thereby promoting the formation of amorphous structures. It is worth noting that this trend is more pronounced during the processing of octahedral abrasives with higher sharpness, reflecting a greater number of fractures of Si-C bonds, Si-SI/C-C bonds, and distant Si-C bonds during grinding, a higher density of crystal defects, and a further reduction in structural orderliness.

**Figure 17:**
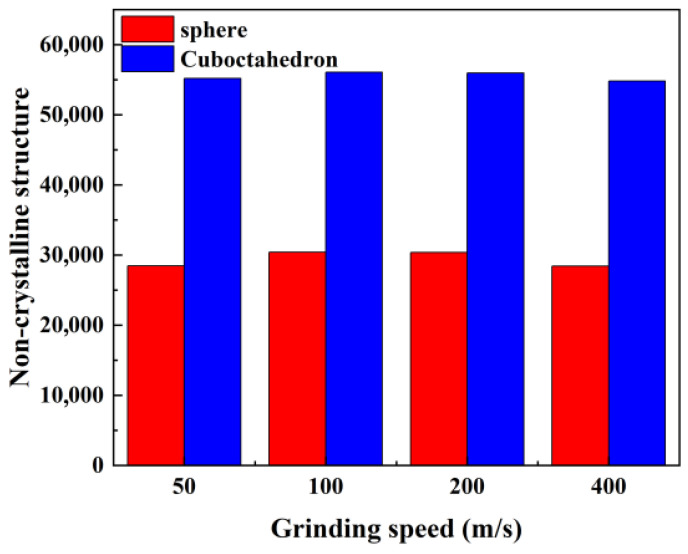
Number of amorphous atomic structures under different conditions.

Crystal structure types can be identified by methods such as CAN and IDS [37]. The number of amorphous atomic structures under different conditions is shown in Figure 17. During the grinding process, regardless of the sharpness of the abrasive, both the cubic diamond structure and the hexagonal diamond structure in the 4H-SiC workpiece will gradually transform into an amorphous state due to the breaking of chemical bonds, and the number of amorphous atoms will increase accordingly. However, the effect of grinding speed on the degree of amorphous shows nonlinear characteristics: with the increase in grinding speed, the number of amorphous atoms first increases and then decreases, reaching a peak near 100 m/s. This indicates that the sharpness of the abrasive and the grinding speed jointly regulate the process and degree of crystal structure transformation.

### 3.5. Abrasive Research

**Figure 18:**
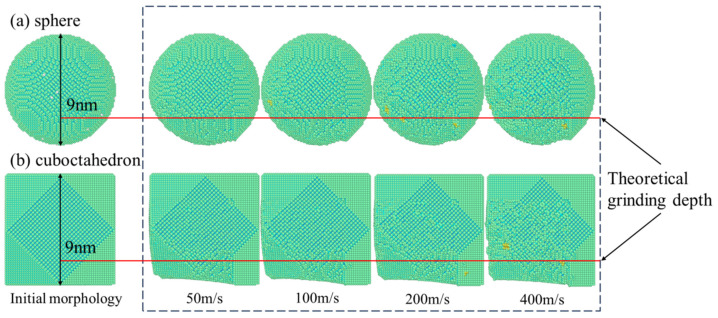
Abrasive wear morphology. (**a**) sphere; (**b**) cuboctahedron.

The removal behavior of workpiece material is jointly influenced by grinding speed and abrasive wear. In the interaction between diamond abrasives and 4H-SiC workpieces, the abrasives themselves will also undergo compression, deformation and wear processes. The evolution of wear morphology of two types of diamond abrasives with different sharpness under different grinding speeds is shown in Figure 18. As can be seen from the figure, with the increase in grinding speed, the abrasive surfaces of both shapes show more significant wear and deformation, especially under high-speed conditions. This indicates that high-speed grinding intensifies the interaction between the abrasive and the workpiece, thereby accelerating the wear of the abrasive. In particular, octahedral abrasives, due to their sharp geometric features, are more prone to local wear at the edges and corners.

**Figure 19:**
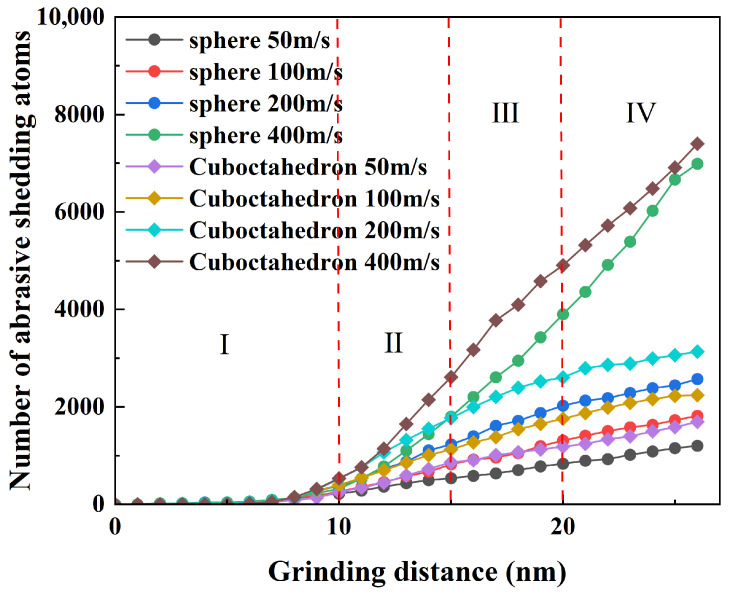
Number of atoms shed due to abrasive wear.

Figure 19 further statistically analyzes the changing trend of the number of atoms worn off by diamond abrasives under different sharpness and grinding speeds. In the figure, the grinding process is divided into four stages (I, II, III, and IV) by red dotted lines, and the wear characteristics of each stage are different. Under the same grinding conditions, the number of atoms shed by regular octahedral abrasives is generally higher than that of spherical abrasives. As the grinding distance increases, the number of wear atoms of the abrasive shows an upward trend under all working conditions, reflecting that the wear gradually accumulates with the grinding process. At lower grinding speeds (50 m/s, 100 m/s and 200 m/s), the number of wear atoms of the two types of abrasives is relatively small; in stages I and II, the wear increases rapidly due to initial contact, while in stages III and IV, the growth tends to be moderate. However, when the grinding speed is further increased, the number of wear atoms of both abrasives rises significantly, especially for the regular octahedral abrasive, which is more obvious. This indicates that under high-speed grinding conditions, octahedral abrasives are more prone to continuous wear, and their wear rate does not show a significant decrease until the end of the simulation.

**Figure 20:**
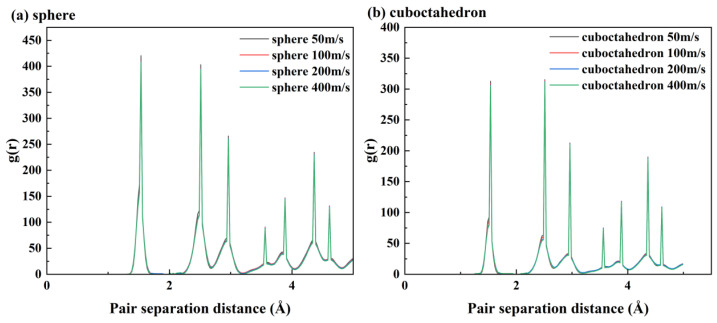
RDF of diamond after grinding at different grinding speeds. (**a**) sphere; (**b**) cuboctahedron.

To study the structural evolution of the diamond abrasives during grinding, the radial distribution function of their carbon atom pairs was calculated, and the results are shown in Figure 20. In graphite crystals, the bond length of adjacent sp^2^ hybrid carbon atoms within the same layer is 1.42 Å, and the bond angle is 120°. The bond lengths and bond angles of the nearest carbon atoms in diamond crystals are 1.54 Å and 109.5°, respectively. The energy minimization process in molecular dynamics simulation causes slight shifts in bond length and bond angle at peak positions, with deviations of approximately 0.1 Å and 2.5°, respectively. It can be observed from Figure 20 that a new peak appears near 1.46 Å and the peak intensity weakens at 1.52 a, indicating that the surface of the diamond abrasive undergoes graphitization transformation during grinding.

**Figure 21:**
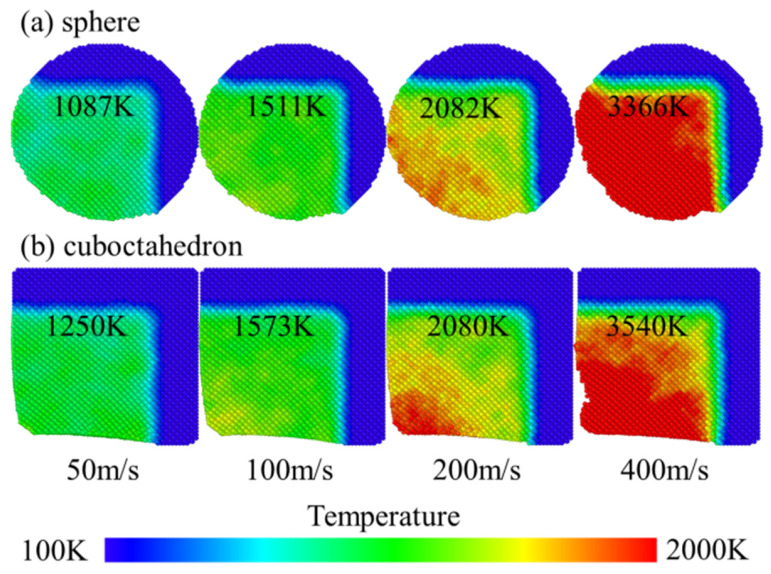
Temperature distribution and maximum temperature of the abrasive after grinding at different grinding speeds. (**a**) sphere; (**b**) cuboctahedron.

**Figure 22:**
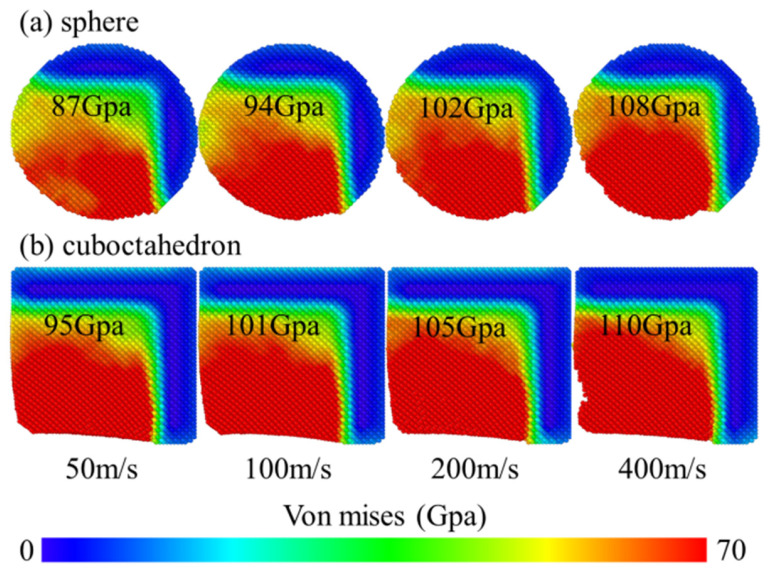
Stress distribution and the maximum stress magnitude of the abrasive after grinding at different grinding speeds. (**a**) sphere; (**b**) cuboctahedron.

The interaction between 4H-SiC workpieces and diamond abrasives can cause significant changes in the local temperature and stress levels of the abrasives, and high temperature and high stress are considered to be two key factors inducing the transformation of diamond to graphite [38,39,40]. Therefore, it is necessary to conduct a systematic analysis of the distribution and magnitude of the temperature field and stress field of diamond abrasive during the grinding process. Previous studies have pointed out that when the wear temperature of diamond abrasive is lower than 1910 K, its graphitization process is mainly driven by shear stress, and the critical stress for the transformation of diamond to graphite is approximately 95 GPa [41]. The temperature and stress distribution of diamond abrasive under different grinding speeds, as well as the changes in their extreme values, are shown in Figure 21 and Figure 22, respectively. It can be observed that both the high-temperature zone and the high-stress zone are concentrated at the wear edge of the abrasive and gradually expand inward as the grinding process progresses. As the grinding speed increases, the overall temperature of the abrasive rises, and the maximum stress value at the edge also increases accordingly. Especially under the same working conditions, the maximum temperature and maximum stress generated by octahedral abrasives both exceed those of spherical abrasives. The simulation results (Figure 21) show that at lower grinding speeds (50 m/s and 100 m/s), the overall temperature of the abrasive has difficulty reaching 1910 K, which can trigger large-scale graphitization. However, even at these speed conditions, the local maximum stress at the abrasive edge has exceeded the graphitization critical stress of 95 GPa (Figure 22). This indicates that in the early stage of grinding or at lower speeds, the graphitization of diamond abrasives may be mainly driven by stress rather than dominated by thermal activation.

**Figure 23:**
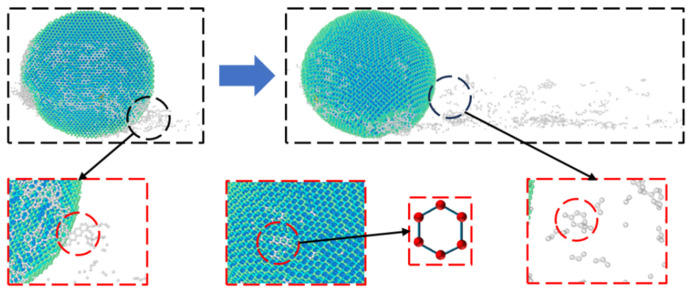
Passivation, wear and graphitization of diamond at different grinding distances.

The morphological evolution of diamond abrasive during its movement along the grinding direction is shown in Figure 23. Obvious abrasive grain shedding and size reduction can be observed from the grinding trajectory, indicating that the abrasive has undergone significant wear. Previous studies have indicated that a temperature of approximately 800 K is sufficient to weaken the C-C bonds in diamond and promote the separation of carbon atoms from the lattice [42]. As can be seen from Figure 18, the overall size of the diamond abrasive decreases after grinding, especially the sharp tips of the regular octahedral abrasive show obvious passivation, which is a direct manifestation of the friction and wear effect. Figure 19 further shows that a large number of carbon atoms fall off the abrasive surface at different speeds, which is in line with the wear characteristics of the abrasive during the plowing process. The coordination numbers of carbon atoms in diamond and graphite are 4 and 3 respectively, corresponding to different hybridization states (sp^3^ and sp^2^) and spatial arrangements. sp^2^ hybrid atoms with graphite characteristics can be detected in the surface layer and shed atoms of deformed diamond abrasives. In addition, a single six-ring structure similar to a graphite six-membered ring was also observed in the abrasive wear area.

Based on the above, the wear of diamond abrasive particles during the nanomilling process of 4H-SiC is not merely a simple mechanical atomic peeling, but rather a result of the combined effect of stress/heat-induced graphitization and mechanical wear. As shown in Figure 21 and Figure 22, even at a relatively low milling speed (50 m/s), the local von Mises stress at the edge of the abrasive particles has exceeded the critical value of 95 GPa for graphitization, causing the diamond lattice to transform into graphite. The interlayer bonding force of the graphite phase is much weaker than that of the covalent bonds in diamond, making this area prone to atomic detachment under subsequent frictional forces. As the milling speed increases, the thermal activation effect intensifies, and the graphitization rate accelerates, further exacerbating the wear of the abrasive particles. Therefore, graphitization is the key pre-requisite mechanism that initiates and intensifies the wear of diamond abrasive particles, rather than being a secondary phenomenon that accompanies wear.

### 3.6. Surface Roughness

**Figure 24:**
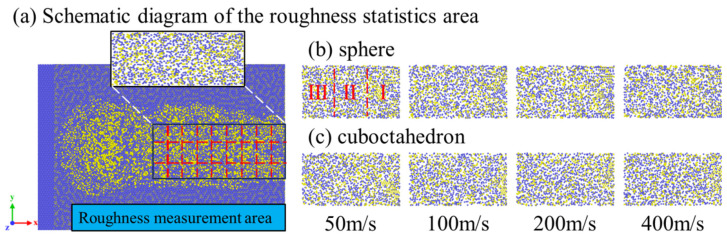
Top view of the surface atoms of the workpiece under different grinding conditions. (**a**) Schematic diagram of the roughness statistics area; (**b**) sphere; (**c**) cuboctahedron.

To systematically evaluate the influence of abrasive sharpness and grinding speed on the surface roughness of 4H-SiC workpieces, and to explore the mechanism of abrasive wear and graphitization on surface morphology, this study aims to reveal how abrasive characteristics and process conditions jointly regulate the surface quality of workpieces. Through comparative analysis, it is expected to provide a theoretical basis for optimizing grinding parameters and abrasive selection, thereby enhancing the integrity of the machined surface. The surface roughness calculation was carried out by referring to the method of Wang et al. [43]; the roughness range of the simulation results in their study was from 0.87 Å to 2.19 Å. As shown in Figure 24a, a rectangular area (black box) with a length of approximately 15 nm and a width of approximately 8 nm was selected on the surface of the 4H-SiC workpiece, and the z-coordinates of the surface atoms above the red dotted line inside it were extracted as the calculation samples. The selection of this area is based on the following considerations: The total grinding distance of the diamond abrasive on the workpiece surface is approximately 25 nm. However, in the later stage of grinding, the continuous pressing of the abrasive causes the surface atoms of the workpiece to be unable to fully recover elastically, and its Z coordinate value is lower than the true surface height, introducing systematic errors. To avoid this influence, the roughness calculation is only for the sections where the material removal in the early stage of grinding is relatively stable. Based on the Z-coordinate data of the selected atoms, the surface roughness Ra is calculated by the following formula:

(1) $$Ra=\frac{\sum |z_{i}-\bar{z}|}{n}$$

Here $z_{i}$, $\bar{z}$ and *n* respectively represent the coordinates of the *i*-th surface atom, the average coordinate, and the total number of atoms in the region. Figure 24b,c show the top view profiles of surface atoms of 4H-SiC workpieces processed with diamond abrasives of different sharpness at different grinding speeds. In the figure, the workpiece surface is divided into three zones along the grinding direction: Zone I (0–5 nm), Zone II (5–10 nm), and Zone III (10–15 nm), corresponding respectively to the pre-, mid-, and post-grinding stages of the grinding process, in order to analyze the evolution of the surface morphology in more detail.

**Figure 25:**
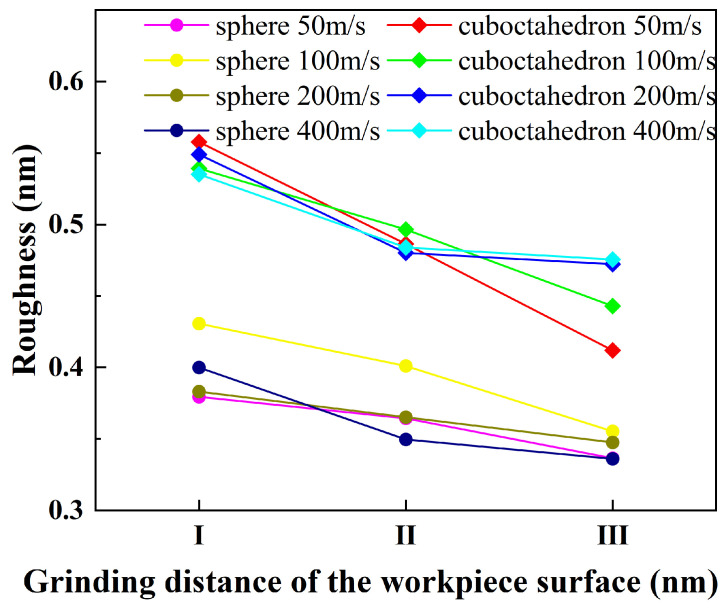
Influence of different grinding conditions on the surface roughness of workpieces.

The influence of two different sharpness levels of diamond abrasives on the surface roughness of 4H-SiC workpieces under different grinding speeds is shown in Figure 25. In the figure, the roughness performance of spherical abrasives and regular octahedral abrasives at various grinding speeds is distinguished by different colors and markings. The surface roughness Ra value is used to quantify the degree of microscopic unevenness on the surface. The smaller the value, the smoother the surface.

The results show that at the same grinding speed, there are significant differences in the influence of spherical abrasives and regular octahedral abrasives on the surface roughness of workpieces: the surface roughness produced by regular octahedral abrasives is generally higher than that of spherical abrasives. This is mainly due to the sharp geometric features of the octahedral abrasive grains. During the grinding process, their contact behavior with the workpiece surface is more complex, which is prone to introduce more microscopic undulations and irregularities. With the increase in grinding speed, the Ra value of the workpiece surface roughness shows a downward trend under most conditions. Higher speed helps to enhance the material removal efficiency, thereby reducing the residual micro-protrusions and defects on the surface and improving surface uniformity. To analyze the evolution of roughness along the grinding direction, the workpiece surface is divided into three regions (I, II, and III). In the initial grinding stage (Zone I), the abrasive wear is slight, the sharpness is well maintained, the grinding temperature and stress level are relatively low, and the graphitization has not significantly occurred. At this time, the contact area between the abrasive and the workpiece is the largest, and the surface roughness of the workpiece reaches its peak. As grinding proceeds (in Zones II and III), the abrasive gradually wears out (see the blunting phenomenon at the edge of the octahedral abrasive in Figure 18), and the diamond surface undergoes graphitization transformation, resulting in a decrease in abrasive hardness and a reduction in the actual contact area. In this case, even if the grinding depth remains unchanged, the roughness change in the workpiece surface tends to be gentle.

As can be seen from the above, the sharpness of the abrasive and the grinding speed jointly regulate the evolution of the surface roughness of the workpiece. In the early stage of grinding, the large contact area of the sharp abrasive leads to a significant increase in surface roughness. With the development of abrasive wear and graphitization, the contact area decreases, the hardness of the abrasive decreases, and the growth of surface roughness gradually slows down.

## 4. Conclusions

In this study, through molecular dynamics simulation, the grinding process of 4H-SiC crystals by diamond abrasive grains of different sharpness at different grinding speeds was systematically analyzed. The surface morphology evolution, material removal behavior, subsurface damage mechanism and microstructure transformation were mainly investigated, and the coupling relationship between temperature field, stress field, abrasive wear and the graphitization phenomenon was explored. The main conclusions are as follows:

(1) The sharpness of abrasive grains and grinding speed jointly regulate the material removal efficiency and surface morphology. Octahedral abrasive grains, with their sharp geometric features, have a higher material removal rate, but they are also prone to form deeper grooves and steeper side walls on the workpiece surface, affecting surface integrity.
(2) Grinding speed is a key factor affecting subsurface damage. Octahedral abrasive grains cause higher local temperatures and stresses during processing, leading to more significant subsurface damage. With the increase in grinding distance, the thermodynamic coupling effect intensifies, and the high-temperature and high-stress zone expands. Increasing the grinding speed will intensify the strain rate and shear deformation, causing the dislocation evolution to lag behind the material deformation and further promoting temperature rise and stress concentration. By reasonably increasing the grinding speed, the depth of subsurface damage can be suppressed to a certain extent, and the optimization of processing quality can be achieved.
(3) The material removal behavior is subject to the combined effect of grinding speed and abrasive wear. Abrasive wear and graphitization are the main failure mechanisms of diamond abrasive grains in nano-grinding. As the grinding speed increases, the wear of abrasive grains intensifies. Regular octahedral abrasive grains are more prone to wear due to their geometric characteristics. High temperature and high stress jointly drive the transformation of diamond into graphite. Even at a relatively low grinding speed, the local stress at the edge of the abrasive grains can still exceed the critical value of graphitization, inducing structural transformation.
(4) The surface roughness is significantly affected by the sharpness of the abrasive grains and the grinding speed. In the early stage of grinding, the sharp abrasive grains and the large contact area cause the surface roughness to rise rapidly. With the development of abrasive wear and graphitization, the contact area decreases, the hardness of the abrasive particles drops, and the growth of roughness tends to level off.

## Figures and Tables

**Table 1:** Simulation parameter settings.

Parameter	Value
Workpiece material	Single-crystal 4H-SiC
Abrasive materials	Single-crystal diamond
Workpiece size	30 nm × 20 nm × 10 nm
The number of atoms in the workpiece	591,920
The number of abrasive atoms on the sphere	67,661
The number of atoms in a regular octahedral abrasive	107,950
Initial temperature	300 K
Grinding distance	26 nm
Grinding speed	50, 100, 200, 400 m/s
Grinding depth	2 nm
Time step	1 fs
Potential function	Tersoff

## Data Availability

Data will be made available on request.
